# LDCT uptake and determinants of lung cancer screening in Asia: a systematic review and meta-analysis

**DOI:** 10.3389/fpubh.2025.1751146

**Published:** 2026-02-02

**Authors:** Yue Hu, Yuntong Zhao, Pei Dong, Wuqi Qiu, Ayan Mao

**Affiliations:** 1 Institute of Medical Information & Library, Chinese Academy of Medical Sciences, Beijing, China; 2 Peking Union Medical College, Beijing, China

**Keywords:** cancer screening, determinants, LDCT uptake, lung cancer, meta-analysis

## Abstract

**Importance:**

Low-dose computed tomography (LDCT) lung cancer screening (LCS) has been shown to significantly reduce mortality rates. As its effectiveness relies on LDCT uptake, understanding uptake rates and their determinants can enhance the implementation and effectiveness of screening programs.

**Objective:**

This study aimed to analyze LDCT uptake and its influencing factors in lung cancer screening within an Asian population.

**Methods:**

Studies published between 1 January 2011 and 31 October 2025 were retrieved from four databases, and those reporting LDCT uptake and/or the factors influencing it were included. A random-effects model was applied to combine the effect estimates and 95% confidence intervals. Subgroup analyses were conducted to explore heterogeneity.

**Results:**

A total of 35 studies involving 1,716,756 participants were analyzed, yielding a pooled LDCT uptake rate for lung cancer screening of 46% (95% confidence interval [CI], 41–51%). Program-level factors included sample scale, year of LDCT uptake, and program setting (*p* < 0.05). Patient-level factors that facilitated participation included a family history of lung cancer (odds ratio [OR], 1.95; 95%CI, 1.45–2.63), harmful occupational exposure (OR, 1.48; 95%CI, 1.33–1.64), chronic respiratory diseases (OR, 1.97; 95%CI, 1.62–2.38), alcohol consumption (OR, 1.20; 95%CI, 1.06–1.36), passive smoking exposure (OR, 1.43; 95%CI, 1.24–1.64), a higher body mass index (BMI; OR, 1.12; 95%CI, 1.05–1.20), and higher education levels (OR, 1.35; 95%CI, 1.17–1.56). Patient-level barriers included being a man (OR, 0.61; 95%CI, 0.55–0.68), engaging in frequent exercise (OR, 0.89; 95%CI, 0.84–0.94), smoking (OR, 0.76; 95%CI, 0.66–0.88), and being middle-aged (OR, 0.92; 95%CI, 0.85–0.99).

**Conclusion:**

LDCT uptake for lung cancer screening is lower in Asia than in academic programs, and it varies widely due to program design and population characteristics. Adopting smaller-scale screening designs and targeting key populations may help improve implementation efforts.

**Systematic review registration:**

https://www.crd.york.ac.uk/PROSPERO/view/CRD42025641277, identifier CRD42025641277.

## Introduction

Lung cancer is the second most prevalent cancer and the leading cause of cancer-related deaths globally (1, 2). With the highest incidence and mortality rates among Asian men and East Asian women, lung cancer presents a considerable disease burden on populations across Asia (2).

Early detection of lung cancer improves prognosis (3). The 5-year survival rate of patients with stage IA is over 77%, while for those with stage IVA, it is only 10% (4). The National Lung Screening Trial (NLST) and the NELSON study have demonstrated that lung cancer screening utilizing low-dose computed tomography (LDCT) significantly reduces mortality from lung cancer (5, 6). Furthermore, various studies have provided evidence supporting the effectiveness of LDCT-based lung cancer screening in reducing mortality in real-world settings (7). Thus, early detection and diagnosis of lung cancer through LDCT screening is now recommended for high-risk populations in many countries.

The effectiveness of lung cancer screening depends on LDCT uptake, and understanding uptake rates and their determinants can improve the implementation efforts. Notably, there is a lack of comprehensive pooled analysis of LDCT uptake for lung cancer screening specifically in Asian populations (8, 9). Therefore, this study aims to estimate the current status of LDCT screening and to identify the factors influencing its uptake within Asian populations. These findings are intended to inform targeted interventions and optimize screening strategies in order to enhance coverage, ultimately contributing to a reduced burden of lung cancer in these populations.

## Methods

The protocol for this systematic review is based on the Cochrane Handbook for Systematic Reviews of Interventions (10) and is registered on PROSPERO (registration number: CRD42025641277). We report the results in accordance with the Preferred Reporting Items for Systematic Reviews and Meta-Analyses (PRISMA) and the Meta-analysis of Observational Studies in Epidemiology (MOOSE) statements (11, 12).

### Search strategy and eligibility criteria

One investigator (YH) systematically searched four databases, namely PubMed, Embase, Cochrane, and Web of Science, with no language restrictions. Since the publication of the NLST results in 2011, we considered publication dates from January 1, 2011 to October 31, 2025. The search terms included Medical Subject Headings and free-text terms related to “lung cancer,” “cancer screening,” “LDCT,” “Asia,” and “participation.” The full search strategy is presented in Supplementary Table S1. The electronic database search was supplemented with manual searches of reference lists from related review articles regarding lung cancer screening in Asia. We included studies that reported LDCT uptake rates in the first round of screening and/or identified determinants of lung cancer screening in Asia. We included cohort and cross-sectional studies that conducted screening programs for high-risk populations. We also considered studies reporting on screening programs using only LDCT procedures for the first round. The specific procedure for the first LDCT involves assessing high-risk individuals through questionnaires or other methods; those identified as high-risk or eligible then undergo lung cancer screening. Studies that utilized LDCT in combination with chest X-rays or sputum cytology were excluded. Gray literature, theses/dissertations, and national registry reports were excluded. We also excluded randomized controlled trials, reviews, conference abstracts, guidelines, letters, and commentaries, which lacked sufficient data for conducting the meta-analysis.

### Selection and data collection process

EndNote 21 (version 21.0.1) was used as the reference manager. All search results were compiled, and duplicates were removed. Two researchers (YH and YZ) independently screened for citations. The initial screening of titles and abstracts was conducted based on predefined inclusion and exclusion criteria, followed by further screening of the full text of eligible citations. Data extraction was also conducted independently by the two researchers through a full-text review. Any discrepancies between the two researchers were resolved through consensus or consultation with a third researcher (AM), after which the final results were reviewed. For studies that did not report complete data, we did not contact the original authors but assumed that the data were missing and excluded the study.

### Data items

We recorded the number of individuals eligible for lung cancer screening and those who underwent LDCT to calculate the LDCT uptake rate (defined as the number of screened individuals divided by the number of eligible individuals). For eligibility, we considered participants who were eligible for high-risk assessments or program guidelines. For those who underwent LDCT screening, we included participants who were initially screened.

We extracted general study information, which included the first author, publication year, study design, academic or community setting, sample size, recruitment period, country/area, eligibility criteria, and the age range of people entitled to LCS. We also captured the baseline information about the participants and characteristics related to LDCT uptake, such as sex, age, family history of lung cancer, harmful occupational exposure, chronic respiratory disease, drinking status, smoking status, passive smoking exposure, frequent exercise, BMI, and educational level.

### Quality assessment

We used the Newcastle–Ottawa Scale (NOS) for quality assessment (Supplementary Tables S2, S3). The NOS consists of three parts: selection, comparability, and exposure or outcome. The selection component examines whether the selection of study subjects is representative and comparable, with a maximum score of four points. The comparability component assesses the similarity between groups and whether important confounders were controlled for, with a maximum score of two points. The exposure or outcome component evaluates whether the measurement of exposure factors was accurate and whether the assessment of outcomes was objective, with a maximum score of three points. The NOS has a total score of 9 points; to adapt it to the current study, three items were excluded from the assessment; consequently, the maximum possible score was adjusted to 5. Studies with a score of 5 were considered to be of high quality, a score of 3–4 was considered to be of moderate quality, and a score of less than 1–3 suggests that the study was of low quality and may have a high risk of bias.

### Statistical analysis

All statistical analyses were performed using RevMan (version 5.4) and R (version 4.4.2) with the “meta,” “metaprop,” and “forestploter” packages. Effect sizes were reported as LDCT uptake rates with 95% confidence intervals (CIs) and odds ratios (ORs) with 95% CIs for factors influencing LDCT uptake.

Heterogeneity between studies was assessed using the *Q* test and the *I*^2^ statistic. If the *p*-value of the *Q* test was greater than 0.05 and *I*^2^ was less than 50%, it indicated no significant heterogeneity between the studies.

Meta-analysis of rates was conducted using a random-effects model, with weights calculated using the inverse variance method. Data transformation was performed using the Freeman–Tukey double arcsine transformation (PFT) to stabilize variances. The pooled LDCT uptake rate and 95% CI were calculated as weighted averages from the individual studies. For patient-level factors influencing LDCT uptake, pooled odds ratios (ORs) were estimated using a random-effects model and the inverse variance method. For studies providing event counts, the ORs were calculated and then transformed to logORs and standard errors of logORs (selogORs) to calculate the pooled OR.

Subgroup analyses were performed to assess the influence of program- and system-level factors, such as sample scale, year of LDCT uptake, setting, and division of the data collection period on heterogeneity. The year of LDCT uptake was represented by the median of the start and end years of data collection. Given the extended time frame covered by some studies, and the fact that the included studies only reported the first-round outcomes of LDCT uptake, the median year of enrollment was therefore utilized to represent the average time of initial uptake for screening. The data collection period was calculated by subtracting the recruitment start date from the end date. The study setting was classified as either academic or community, while the sample size was categorized into different ranges.

Publication bias was assessed using the Egger test, where a *p*-value of <0.05 indicates the presence of publication bias, and a p-value of ≥0.05 suggests no publication bias. LDCT uptake rate and its standard error (SE) were log-transformed into log (proportion) and selog (proportion), respectively, before conducting the Egger test in order to minimize Type I error due to large sample sizes.

## Results

**Figure 1:**
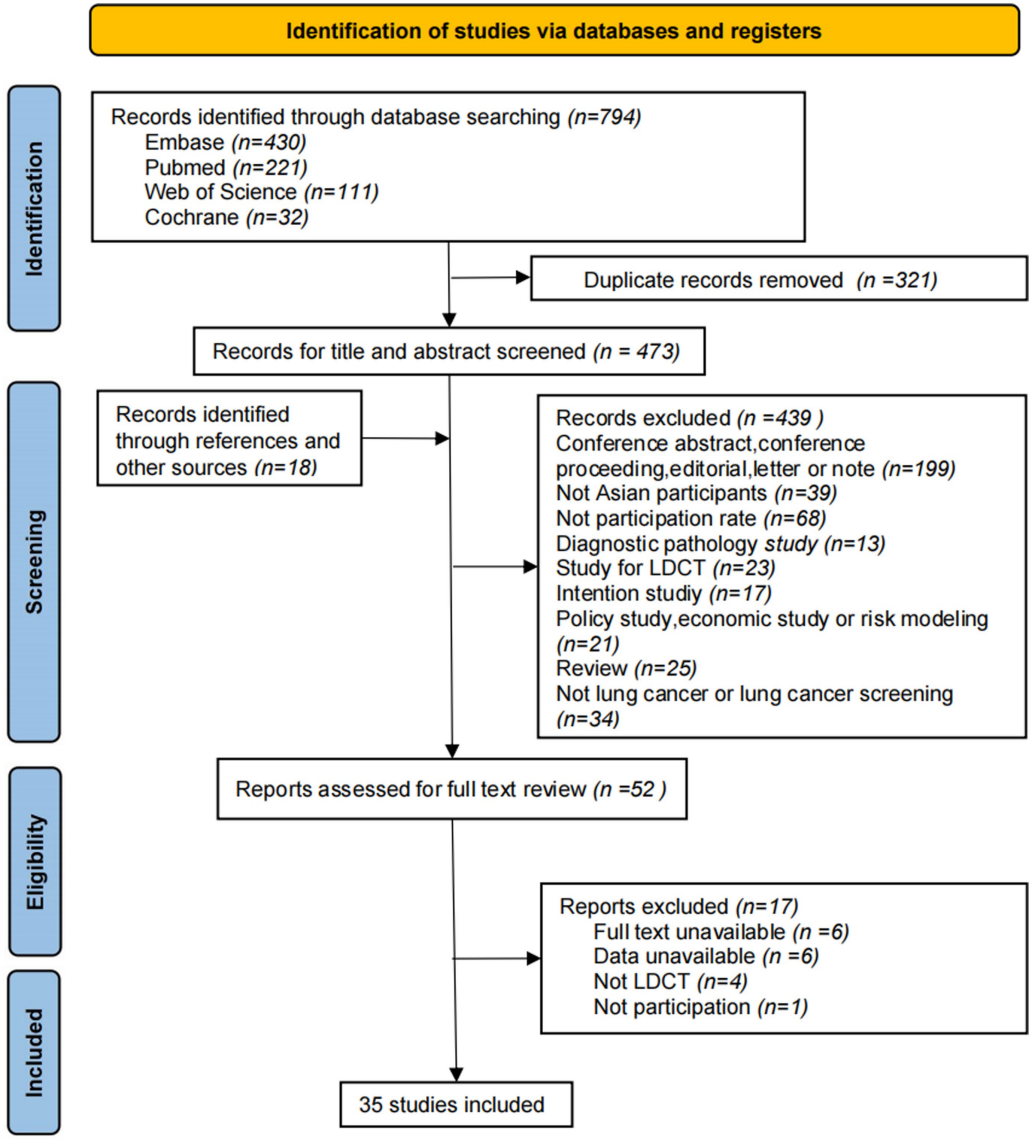
PRISMA flow diagram of study selection.

A total of 794 articles were retrieved from the databases. After removing 321 duplicates, an additional 18 citations from other sources were included. In total, 491 titles and abstracts were screened, and 439 studies were excluded based on the predefined criteria. After a full-text review of 52 articles, 35 studies, and 1,716,756 patients were ultimately included (Figure 1). According to the NOS scale, 14 studies were assessed as high-quality and 20 as moderate-quality, with only one study rated as low-quality. Overall, the quality of the included studies ranged from moderate to high. The results of the Egger test showed a *p*-value of >0.05, indicating no significant publication bias.

All studies were published between 2016 and 2025. There were 14 cross-sectional studies, 20 prospective cohort studies, and one retrospective study. The majority of the studies were conducted in China (*n* = 32), followed by South Korea (*n* = 2) and Kazakhstan (*n* = 1). A total of 30 studies were conducted in community settings, and five studies took place in academic settings. The data collection periods for the studies ranged from 1 to 7 years. In terms of eligibility assessment for the screening populations, 29 studies used the Cancer Screening Program in Urban China (CanSPUC) risk score system, four studies followed the eligibility criteria outlined in the guidelines for lung cancer screening, and two studies used specified assessment methods based on programs. Detailed information is provided in Table 1.

**Table 1:** Characteristics of the included studies.

Source	Total	Study type	Setting	Definition of LDCT uptake	Data collection period	Risk assessment/eligible	the age range of people entitled to LCS	Country
A. Panina et al. (50)	5000	cross-sectional	academic	uptake LDCT/eligible for program	2018.06-2020.05	program criteria 1 ^a^	40-75 years	Kazakhstan
C. Wang et al. (51)	3376	cross-sectional	community	uptake LDCT/high risk	2020.06-2020.11	the CanSPUC risk score system ^b^	40-74 years	China
D.H. Wei et al. (52)	12449	prospective cohort	community	uptake LDCT/high risk	2014-2016	the CanSPUC risk score system	40-74 years	China
D. Liang et al. (53)	11052	prospective cohort	community	uptake LDCT/high risk	2018.11-2019.05	the CanSPUC risk score system	40-74 years	China
D. Liang et al. (54)	54846	prospective cohort	community	uptake LDCT/high risk	2013-2019	the CanSPUC risk score system	40-74 years	China
F. Zeng et al. (55)	9139	cross-sectional	community	uptake LDCT/high risk	2018.11-2020.10	the CanSPUC risk score system	40-74 years	China
H. Du et al. (56)	36790	prospective cohort	community	uptake LDCT/high risk	2014-2018	the CanSPUC risk score system	40-74 years	China
H.F. Xiao et al. (57)	12789	cross-sectional	community	uptake LDCT/high risk	2017-2018	the CanSPUC risk score system	40-74 years	China
H. Xiao et al. (58)	56173	prospective cohort	community	uptake LDCT/high risk	2012-2018	the CanSPUC risk score system	40-74 years	China
J. Du et al. (59)	41183	prospective cohort	community	uptake LDCT/high risk	2012-2017	the CanSPUC risk score system	40-74 years	China
J. Lee et al. (60)	400	cross-sectional	academic	uptake LDCT/eligible for program	2016.11-2017.03	Korean guidelines for lung cancer screening ^c^	55-74 years	Korea
J. Pan et al. (61)	2529	cross-sectional	academic	uptake LDCT/general people	2022.08-2022.09	Combing the criteria of China guidelines and the “Consensus on Lung Cancer Screening and Management in China” ^d^	50-74 years	China
J. Ren et al. (62)	5220	cross-sectional	community	uptake LDCT/high risk	2018-2019	the CanSPUC risk score system	40-74 years	China
J.Y. Zhu et al. (63)	11512	cross-sectional	community	uptake LDCT/high risk	2015	the CanSPUC risk score system	40-74 years	China
L.W. Guo et al. (64)	55428	cross-sectional	community	uptake LDCT/high risk	2013.10-2019.10	the CanSPUC risk score system	40-74 years	China
L. Wang et al. (65)	24301	prospective cohort	community	uptake LDCT/high risk	2013-2019	the CanSPUC risk score system	40-74 years	China
L. Yang et al. (66)	20371	prospective cohort	community	uptake LDCT/high risk	2014-02019	the CanSPUC risk score system	40-74 years	China
M.J. Liu et al. (67)	16020	cross-sectional	community	uptake LDCT/high risk	2019-2023	the CanSPUC risk score system	40-74 years	China
N. Li et al. (68)	223302	prospective cohort	community	uptake LDCT/high risk	2013.02-2018.10	the CanSPUC risk score system	40-74 years	China
Q. Yang et al. (69)	18508	prospective cohort	community	uptake LDCT/high risk	2014-2019	the CanSPUC risk score system	40-74 years	China
S.L. Zhao et al. (70)	51703	retrospective cohort	community	uptake LDCT/high risk	2013-2019	the CanSPUC risk score system	40-74 years	China
T. Tian et al. (71)	19414	prospective cohort	community	uptake LDCT/high risk	2015.01-2019.12	the CanSPUC risk score system	40-74 years	China
Thuy Linh Duong et al. (72)	3557	retrospective cohort	community	uptake LDCT/eligible for program	2020	Korean guidelines for lung cancer screening	55-74 years	Korea
W. Cao et al. (73)	221955	prospective cohort	community	uptake LDCT/high risk	2013-2018	the CanSPUC risk score system	40-74 years	China
W.J. Tao et al. (74)	28728	prospective cohort	academic	uptake LDCT/high risk	2020-2021	program criteria 2 ^f^	40-80 years	China
W.Q. Chen et al. (75)	558480	cross-sectional	community	uptake LDCT/high risk	2013-2017	the CanSPUC risk score system	40-74 years	China
X.Y. Gu et al. (76)	31177	prospective cohort	community	uptake LDCT/high risk	2014-2016	the CanSPUC risk score system	40-74 years	China
X. Zhang et al. (77)	11594	cross-sectional	community	uptake LDCT/high risk	2019-2021	the CanSPUC risk score system	40-74 years	China
Y.J. Li et al. (78)	7936	prospective cohort	academic	uptake LDCT/general people	2017.05-2019.12	NCCN Clinical Practice Guideline^e^	≥50 years	China
Y.P. Lin et al. (79)	31824	prospective cohort	community	uptake LDCT/high risk	2014-2018	the CanSPUC risk score system	40-74 years	China
Y.S. Zhang et al. (80)	3589	cross-sectional	community	uptake LDCT/high risk	2020.01-2021.12	the CanSPUC risk score system	40-74 years	China
Y. Wen et al. (81)	17983	cross-sectional	community	uptake LDCT/high risk	2017.10-2018.10	the CanSPUC risk score system	40-74 years	China
Y.Z. Liu et al. (82)	10623	prospective cohort	community	uptake LDCT/high risk	2014-2016	the CanSPUC risk score system	40-74 years	China
Z.F. Yu et al. (83)	39669	prospective cohort	community	uptake LDCT/high risk	2012-2017	the CanSPUC risk score system	40-74 years	China
Z.-Yu et al. (84)	58136	prospective cohort	community	uptake LDCT/high risk	2012-2019	the CanSPUC risk score system	40-74 years	China

^a^ Program criteria 1: Aged 40-75 years; with no history of any cancer or severe comorbid conditions; no history of a CT examination during the year; and a weight of less than 140 kg.

^b^ The CanSPUC risk score system: The Cancer Screening Program in Urban China (CanSPUC) risk score system is a cancer risk assessment system developed based on the Harvard Cancer Risk Index. It incorporates epidemiological data on common cancers in China and employs a consensus-building approach through a multidisciplinary expert panel to identify major cancer risk factors and assign corresponding risk values for Chinese adults.

^c^ Korean guidelines for lung cancer screening: Aged 55−74 years; with a smoking history of at least 30 pack-years; used tobacco within the last 15 years.

^d^ Combing the criteria of China guidelines and the “Consensus on Lung Cancer Screening and Management in China”: Aged 50-74 years; with a smoking history of ≥30 pack-years.

^e^ NCCN Clinical Practice Guideline: Aged ≥50 years; with a smoking history of ≥20 pack-years.

^f^ Program criteria 2: Aged 40-80 years.

**Figure 2:**
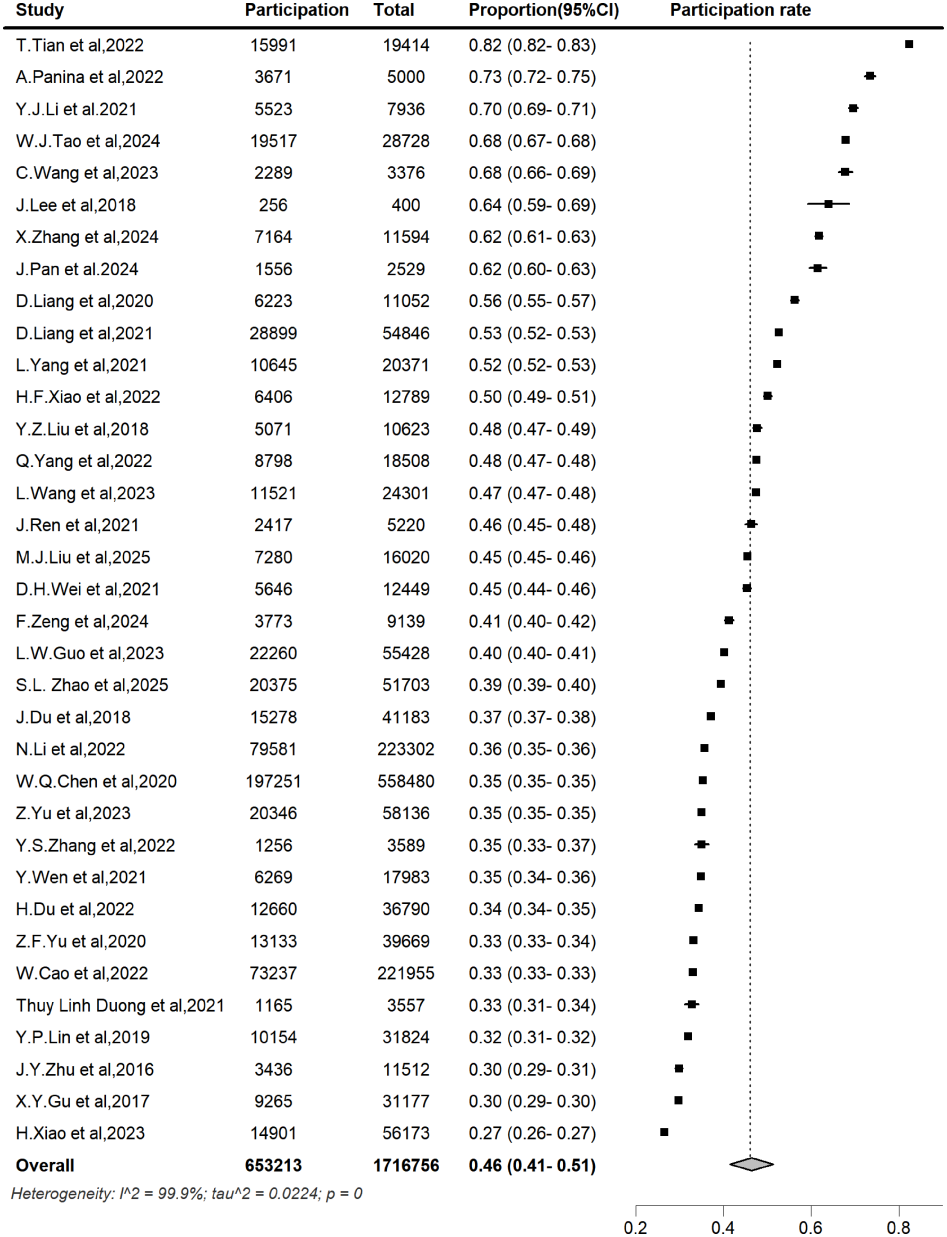
LDCT uptake rate of lung cancer screening in Asia.

The pooled LDCT uptake rate for lung cancer screening across the 35 studies was 46% (95% CI: 41–51%), with individual studies ranging from 27 to 82% (Figure 2).

Subgroup analysis revealed a significant difference in LDCT uptake rates across different uptake-year groups (*p* = 0.0002). The LDCT uptake rate for lung cancer screenings between 2011 and 2015 was 0.35 (95% CI: 0.31–0.39), while the LDCT uptake rate for screenings between 2016 and 2020 was 0.51 (95% CI: 0.44–0.57), and the LDCT uptake rate between 2021 and 2025 was 0.52 (95% CI: 0.37–0.67). Overall, the LDCT uptake rate for screenings conducted between 2016 and 2025 was higher than that for screenings conducted between 2011 and 2015. Notably, a statistically significant difference was also found in LDCT uptake rates based on program scale (sample size) across studies (*p* < 0.0001). The highest LDCT uptake rate was observed in studies with a sample size of 10^2^ (OR, 0.64, 95% CI: 0.59–0.69), followed by 10^3^ (OR, 0.54, 95% CI: 0.42–0.65), 10^4^ (OR, 0.45, 95% CI: 0.39–0.51), and 10^5^ (OR, 0.35, 95% CI: 0.33–0.36). An increase in the sample size and program scale was accompanied by a significant decline in LDCT uptake rates. Subgroup analysis by setting showed significant differences in LDCT uptake rates (*p* < 0.0001). In academic settings, the LDCT uptake rate was 0.68 (95% CI: 0.63–0.72), which was higher than the LDCT uptake rate of 0.43 (95% CI: 0.38–0.48) observed in community settings. Subgroup analysis by data collection period showed no significant difference (*p* = 0.1017).

Among the factors influencing LDCT uptake, the odds ratios (ORs) for sex, frequent exercise, and age were less than 1, indicating that, compared to men, women were less likely to undergo lung cancer screening (OR, 0.61; 95% CI, 0.55–0.68); compared to those with irregular exercise habits, individuals who exercised regularly were less likely to undergo screening (OR, 0.89; 95% CI, 0.84–0.94); compared to older adults, middle-aged individuals were less likely to undergo screening (OR, 0.92; 95% CI, 0.85–0.99); and compared to non-smokers, smokers were less likely to uptake LDCT screening (OR, 0.76; 95% CI, 0.66–0.88). Conversely, the ORs for family history of lung cancer (OR, 1.95; 95% CI, 1.45–2.63), harmful occupational exposure (OR, 1.48; 95% CI, 1.33–1.64), chronic respiratory diseases (OR, 1.97; 95% CI, 1.62–2.38), drinking (OR, 1.20; 95% CI, 1.06–1.36), passive smoking exposure (OR, 1.43; 95% CI, 1.24–1.64), BMI (BMI > 24 vs. 18.5 ≤ BMI < 24) (OR, 1.12; 95% CI, 1.05–1.20), and educational level (OR, 1.35; 95% CI, 1.17–1.56) were greater than 1, indicating that individuals with a family history of lung cancer, harmful occupational exposure, chronic respiratory diseases, alcohol consumption, passive smoking exposure, a higher BMI, and those who have higher education levels were more likely to undergo lung cancer screening. The *p*-value for the pooled ORs of BMI (BMI < 18.5 vs. 18.5 ≤ BMI < 24) was found to be higher than 0.05, suggesting no statistically significant association between LDCT uptake rate and lower BMI. The details are provided in Table 2.

**Table 2:** Patient-level determinants associated with LDCT uptake.

Characteristic	Comparison	Control	No. of studies	OR(95%CI)	*p*-value
Sex	Male	Female	25 studies	0.61 [0.55, 0.68]	*p* < 0.01
Family history of lung cancer	Yes	No	16 studies	1.95 [1.45, 2.63]	*p* < 0.01
Harmful occupational exposure	Yes	No	10 studies	1.48 [1.33, 1.64]	*p* < 0.01
Chronic respiratory diseases	Yes	No	12 studies	1.97 [1.62, 2.38]	*p* < 0.01
Drinking status	Yes	No	9 studies	1.20 [1.06, 1.36]	*p* < 0.01
Frequent exercise	Yes	No	13 studies	0.89 [0.84, 0.94]	*p* < 0.01
Passive smoking exposure	Yes	No	9 studies	1.43 [1.24, 1.64]	*p* < 0.01
BMI	BMI<18.5	18.5 ≤ BMI<24	12 studies	0.89 [0.77, 1.03]	*p* = 0.29
	BMI > 24	18.5 ≤ BMI<24	12 studies	1.12 [1.05, 1.20]	*p* < 0.01
Educational level	≥12 years of education	<12 years of education	15 studies	1.35 [1.17, 1.56]	*p* < 0.01
Smoking status	Current or ever	Never	15 studies	0.76 [0.66, 0.88]	*p* < 0.01
Age	Middle-aged	Elderly	21 studies	0.92 [0.85, 0.99]	*p* = 0.02

## Discussion

Our pooled analysis of 35 studies revealed an overall LDCT uptake rate of 46% (95% CI: 41–51%), while the 2018 Behavioral Risk Factor Surveillance Survey (BRFSS) reported 17.7% of 1,273,013 eligible patients in the United States (13). In 2017, the BRFSS reported that approximately one in eight of the 85,514 respondents undertook lung cancer screening (14). Despite the methodological variations in surveys, differences in LDCT uptake may reflect systematic and sociocultural disparities between Asian and Western countries, including differences in healthcare accessibility, public awareness of lung cancer screening, and implementation strategies of screening programs. There was also considerable variation across different regions of Asia, ranging from 27 to 82%, which may be related to differences in community and hospital mobilization, public education, and service delivery capacity.

**Figure 3:**
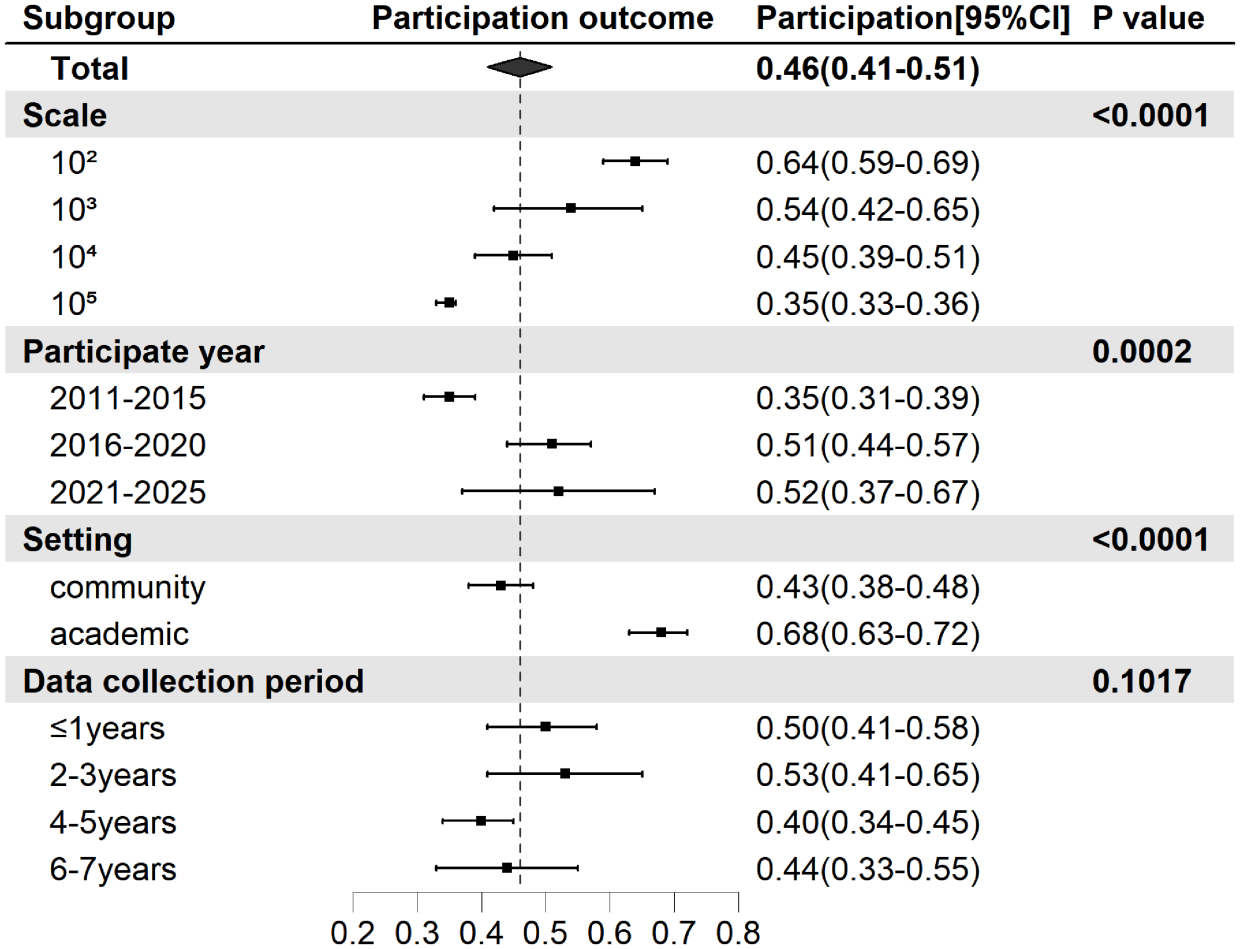
Summarized LDCT uptake by characteristics of screening programs.

This study explored the impact of program design on LDCT uptake (Figure 3). LDCT uptake rates varied significantly across settings, with higher uptake rates observed in academic settings (68% vs. 43% in community settings). This finding aligns with previous research in both Europe and America. In academic programs, the screening rate among high-risk individuals reached 85% (15), and in another academic pilot study, the performance showed an 89% uptake (16). Previous studies have shown that real-world lung cancer screening struggles to achieve the exceptionally high LDCT uptake rates observed in randomized trials, likely due to resource limitations restricting available support, lower willingness to participate among non-trial populations, and less structured coordination or communication between screening program coordinators or physicians and participants (17, 18). Our study reveals a significant association between LDCT uptake rate and sample scale. To our knowledge, this is the first study to directly examine this relationship within lung cancer screening. An inverse relationship was observed between sample size and LDCT uptake rate, with larger sample sizes associated with lower uptake (*p* ≤ 0.0001). Smaller scales (10^2^: 64, 59–69%) demonstrated higher LDCT uptake than larger populations (10^5^: 35, 33–36%). This may be because of the challenges that arise when scaling up the program, such as the dispersion of resources and personnel, which makes it difficult to provide sufficient personalized attention and communication. The lack of individualized services and participant engagement trusts may reduce LDCT uptake rates (19–21). In contrast, smaller sample sizes facilitate centralized management, which enables proper patient selection, follow-up, and adherence that results in high-quality LCS (22). Therefore, a centralized, small-scale screening program is recommended to improve the LCS uptake in regions with adequate service accessibility. In regions with large populations, this approach can be operationalized by establishing multiple screening centers within the same region, each functioning as an independent but highly standardized unit. These centers may be embedded within community hospitals, primary healthcare centers, or community clinics and operate under a unified organizational framework to ensure consistency in protocols, data management, and quality assurance. Each unit delivers a complete screening pathway, including outreach and recruitment, screening implementation, result reporting, and follow-up. By decomposing a large-scale screening initiative into multiple manageable units while maintaining centralized oversight, such a model may facilitate efficient implementation in population-dense areas with adequate service accessibility while preserving process consistency and improving participation rates (16).

LDCT uptake varied markedly across years of uptake (*p* ≤ 0.0001). Since 2011, LDCT uptake rates have steadily increased from 35% (2011–2015) to 52% (2021–2025), potentially reflecting improvements in screening accessibility and the impact of public health campaigns over the past decade.

This study also examined the impact of patient-level characteristics. Individuals with a family history of lung cancer, chronic obstructive pulmonary disease (COPD), occupational exposure, or exposure to passive smoking were more likely to undergo lung cancer screening (23, 24). As these factors are known risk factors for lung cancer (25), these participants may have heightened health awareness and a greater emphasis on self-care. As smoking rates decline, the proportion of lung cancer cases occurring in non-smokers is gradually increasing (26). Individuals with higher educational attainment are more likely to undergo lung cancer screening, which is consistent with previous studies (27, 28). This association may be attributed to a higher level of health awareness, literacy, knowledge, and beliefs (29, 30). Overweight individuals and alcohol drinkers are more likely to undergo lung cancer screening, possibly because overweight or alcohol drinkers often have more chronic conditions, leading to more frequent medical visits and increasing the probability of being recommended for screening. Smokers are less likely to adopt lung cancer screening, consistent with previous studies (31). This may be related to perceived barriers to screening among smokers, such as distrust in their lung cancer risk, fear of a cancer diagnosis, and stigma associated with smoking (32–35). These factors can hinder LDCT uptake during lung cancer screening. Therefore, it is essential to focus on targeted screening awareness and medical support for smokers. Men were less likely to participate in lung cancer screening, which is consistent with previous research findings (36, 37). Future strategies aimed at improving LDCT uptake in lung cancer screening may need to be gender-specific to better address this disparity.

To our knowledge, this systematic review and meta-analysis provides the first comprehensive assessment of uptake rates and associated factors for LDCT-based lung cancer screening across Asian populations. The study provides insights for the implementation of lung cancer screening programs, suggesting a smaller sample size for lung cancer screening. We also explored how participant characteristics affect LDCT uptake, offering guidance on targeting key groups, such as smokers, men, and those with lower education levels, to improve uptake.

This study has several limitations. First, we only included studies conducted in Asia, with a large proportion from China, because of the lack of community-based screening programs in other regions of Asia (38). Second, some unmeasured confounding factors, such as insurance coverage, healthcare service availability, and variations in screening incentives, may have influenced the analysis of LDCT uptake in lung cancer screenings (19, 37, 39–41). Additionally, the impact of socioeconomic factors, such as income level and the ability to pay for medical expenses, on LDCT uptake was also not quantified, which may have led to the omission of key explanatory variables (42–46). Finally, all of the included studies were observational and single-arm studies, which introduced considerable heterogeneity. We used subgroup analyses to explore differences in program characteristics. However, due to limitations in the available data, the study did not conduct a stratified analysis within Asia (such as between East Asia and Southeast Asia), which may have overlooked region-specific issues such as cultural beliefs and differences in policy support (47–49). Given that the heterogeneity between studies was explored through subgroup analyses, and the primary aim of our study was to descriptively summarize LDCT uptake rates and determinants, a meta-regression analysis to quantify the independent contributions of factors was not performed.

## Conclusion

This study provides insights into the uptake of LDCT in lung cancer screening programs across Asia and explores the factors influencing uptake. Currently, real-world uptake of screening in Asia is lower than that observed in academic programs and varies widely, highlighting the need for improvements in program design and increased focus on key populations to enhance the long-term benefits of lung cancer screening programs.

## Data Availability

The original contributions presented in the study are included in the article/Supplementary material; further inquiries can be directed to the corresponding author.
