# Supplementary material for: LDCT uptake and determinants of lung cancer screening in Asia: a systematic review and meta-analysis
Source: Front Public Health. 2026 Feb 2;13:1751146. doi: 10.3389/fpubh.2025.1751146 (PMC12908593; doi:10.3389/fpubh.2025.1751146)
Supplement: Supplementary file 1 [file Data_Sheet_1.docx]

**Supplementary Materials Contents**

**Figure S1.** Funnel plots for meta-analyses of pooled participation rates

**Table S1.** Search strategy

**Table S2.** the Newcastle-Ottawa Scale for risk assessment

**Table S3.** Quality assessment result

**Table S4.** MOOSE checklist

**Table S4.** MOOSE checklist

**Table S5.** List of included studies

| 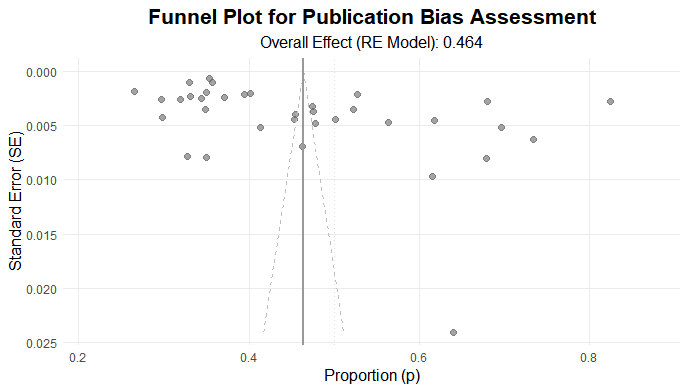 |
| --- |
| **Figure S1.** Funnel plots for meta-analyses of pooled participation rates. |

| **Table S1.** Search strategy. | |
| --- | --- |
| Database | Search strategy |
| PubMed | ("lung neoplasms"[MeSH Terms] OR "neoplasms pulmonary"[All Fields] OR "neoplasm pulmonary"[All Fields] OR "Pulmonary Neoplasm"[All Fields] OR "Pulmonary Neoplasms"[All Fields] OR "neoplasms lung"[All Fields] OR "Lung Neoplasm"[All Fields] OR "neoplasm lung"[All Fields] OR "Lung Cancer"[All Fields] OR "cancer lung"[All Fields] OR "cancers lung"[All Fields] OR "Lung Cancers"[All Fields] OR "Cancer of Lung"[All Fields] OR "Pulmonary Cancer"[All Fields] OR "cancer pulmonary"[All Fields] OR "cancers pulmonary"[All Fields] OR "Pulmonary Cancers"[All Fields] OR "Cancer of the Lung"[All Fields]) AND ("early detection of cancer"[MeSH Terms] OR "Cancer Early Detection"[All Fields] OR "Early Diagnosis of Cancer"[All Fields] OR "Cancer Early Diagnosis"[All Fields] OR "Cancer Screening"[All Fields] OR "screening cancer"[All Fields] OR "Cancer Screening Tests"[All Fields] OR "Cancer Screening Test"[All Fields] OR "screening tests cancer"[All Fields] OR "test cancer screening"[All Fields] OR "tests cancer screening"[All Fields]) AND ("low-dose computed tomography"[All Fields] OR "low-dose computer tomography"[All Fields] OR "low-dose CT"[All Fields] OR "low-dose radiation CT"[All Fields] OR "low-radiation dose CT"[All Fields] OR "low-dose computed tomography"[All Fields] OR "LDCT"[All Fields]) AND ("asia"[MeSH Terms] OR "asia"[All Fields] OR "asian*"[All Fields] OR ("china"[MeSH Terms] OR "china"[All Fields] OR "china s"[All Fields] OR "chinas"[All Fields]) OR ("chineses"[All Fields] OR "east asian people"[MeSH Terms] OR ("east"[All Fields] AND "asian"[All Fields] AND "people"[All Fields]) OR "east asian people"[All Fields] OR "chinese"[All Fields]) OR ("korea"[MeSH Terms] OR "korea"[All Fields] OR "korea s"[All Fields] OR "koreas"[All Fields]) OR "korean*"[All Fields] OR ("japan"[MeSH Terms] OR "japan"[All Fields] OR "japan s"[All Fields] OR "japans"[All Fields]) OR ("east asian people"[MeSH Terms] OR ("east"[All Fields] AND "asian"[All Fields] AND "people"[All Fields]) OR "east asian people"[All Fields] OR "japanese"[All Fields] OR "japaneses"[All Fields]) OR "singapore*"[All Fields] OR ("india"[MeSH Terms] OR "india"[All Fields] OR "india s"[All Fields] OR "indias"[All Fields]) OR "indian*"[All Fields]) AND ("compliances"[All Fields] OR "patient compliance"[MeSH Terms] OR ("participant"[All Fields] OR "participant s"[All Fields] OR "participants"[All Fields] OR "participate"[All Fields] OR "participated"[All Fields] OR "participates"[All Fields] OR "participating"[All Fields] OR "participation"[All Fields] OR "participations"[All Fields] OR "participative"[All Fields] OR "participator"[All Fields] OR "participators"[All Fields]) OR ("adherance"[All Fields] OR "adhere"[All Fields] OR "adhered"[All Fields] OR "adherence"[All Fields] OR "adherences"[All Fields] OR "adherent"[All Fields] OR "adherents"[All Fields] OR "adherer"[All Fields] OR "adherers"[All Fields] OR "adheres"[All Fields] OR "adhering"[All Fields])) AND 2011/01/01:2025/10/31[Date - Publication] |
| Cochrane | (("Neoplasm, Pulmonary"):ti,ab,kw OR ("Lung Neoplasm"):ti,ab,kw OR ("Pulmonary Neoplasms"):ti,ab,kw OR ("Neoplasms, Lung"):ti,ab,kw OR ("Neoplasm, Lung"):ti,ab,kw OR ("Pulmonary Neoplasm"):ti,ab,kw OR ("Neoplasms, Pulmonary"):ti,ab,kw OR ("Lung Cancers"):ti,ab,kw OR ("Cancer of the Lung"):ti,ab,kw OR ("Pulmonary Cancers"):ti,ab,kw OR ("Lung Cancer"):ti,ab,kw OR ("Cancers, Lung"):ti,ab,kw OR ("Cancer, Lung"):ti,ab,kw OR ("Cancer of Lung"):ti,ab,kw OR ("Cancers, Pulmonary"):ti,ab,kw OR ("Cancer, Pulmonary"):ti,ab,kw OR ("Pulmonary Cancer")) AND (("Test, Cancer Screening"):ti,ab,kw OR ("Tests, Cancer Screening"):ti,ab,kw OR ("Screening Test, Cancer"):ti,ab,kw OR ("Cancer Screening Tests"):ti,ab,kw OR ("Cancer Screening Test"):ti,ab,kw OR ("Screening Tests, Cancer"):ti,ab,kw OR ("Cancer Early Detection"):ti,ab,kw OR ("Cancer Early Diagnosis"):ti,ab,kw OR ("Early Diagnosis of Cancer"):ti,ab,kw OR ("Screening, Cancer"):ti,ab,kw OR ("Cancer Screening")) AND ((low-dose computed tomography):ti,ab,kw OR ("low-dose computer tomography"):ti,ab,kw OR ("low-dose CT"):ti,ab,kw OR ("low-dose radiation CT"):ti,ab,kw OR ("low-radiation dose CT"):ti,ab,kw OR ("low-dose computed tomography"):ti,ab,kw OR ("LDCT"):ti,ab,kw) AND ((Asia):ti,ab,kw OR (Asian*):ti,ab,kw OR (China):ti,ab,kw OR (Chinese):ti,ab,kw OR (Korea):ti,ab,kw OR (Korean*):ti,ab,kw OR (Japan):ti,ab,kw OR (Japanese):ti,ab,kw OR (Singapore*):ti,ab,kw OR (India):ti,ab,kw OR (Indian*):ti,ab,kw) AND ((participation):ti,ab,kw OR (participants):ti,ab,kw OR (participate):ti,ab,kw)with Cochrane Library publication date from Jan 2011 to Oct 2025 |
| Embase | ('broncho-pulmonary cancer'/exp OR 'broncho-pulmonary cancer' OR 'bronchopulmonary cancer'/exp OR 'bronchopulmonary cancer' OR 'ca lung'/exp OR 'ca lung' OR 'cancer of the lung'/exp OR 'cancer of the lung' OR 'cancer, lung'/exp OR 'cancer, lung' OR 'carcinogenesis of the lung'/exp OR 'carcinogenesis of the lung' OR 'lung malignancies'/exp OR 'lung malignancies' OR 'lung malignancy'/exp OR 'lung malignancy' OR 'malignancies of the lung'/exp OR 'malignancies of the lung' OR 'malignancy of the lung'/exp OR 'malignancy of the lung' OR 'malignant lung neoplasm'/exp OR 'malignant lung neoplasm' OR 'malignant lung tumor'/exp OR 'malignant lung tumor' OR 'malignant neoplasm of the lung'/exp OR 'malignant neoplasm of the lung' OR 'malignant tumor of the lung'/exp OR 'malignant tumor of the lung' OR 'pulmonary cancer'/exp OR 'pulmonary cancer' OR 'pulmonary malignancies'/exp OR 'pulmonary malignancies' OR 'pulmonary malignancy'/exp OR 'pulmonary malignancy' OR 'schneeberg disease'/exp OR 'schneeberg disease' OR 'schneeberg lung disease'/exp OR 'schneeberg lung disease' OR 'lung cancer'/exp OR 'lung cancer') AND ('screening, cancer'/exp OR 'screening, cancer' OR 'cancer screening'/exp OR 'cancer screening' OR 'early detection of cancer'/exp OR 'early detection of cancer' OR 'early cancer diagnosis'/exp OR 'early cancer diagnosis') AND ('computed tomography low-dose scan'/exp OR 'computed tomography low-dose scan' OR 'ldct (low-dose computed tomography)'/exp OR 'ldct (low-dose computed tomography)' OR 'low-dose (ld) computed tomography (ct)'/exp OR 'low-dose (ld) computed tomography (ct)' OR 'low-dose (ld) computed tomography (ld-ct)'/exp OR 'low-dose (ld) computed tomography (ld-ct)' OR 'low-dose computed tomography (ct)'/exp OR 'low-dose computed tomography (ct)' OR 'low-dose computed tomography (ldct)'/exp OR 'low-dose computed tomography (ldct)' OR 'low-dose computer tomography'/exp OR 'low-dose computer tomography' OR 'low-dose computer tomography (ct)'/exp OR 'low-dose computer tomography (ct)' OR 'low-dose computer tomography (ldct)'/exp OR 'low-dose computer tomography (ldct)' OR 'low-dose ct'/exp OR 'low-dose ct' OR 'low-dose dose ct scan'/exp OR 'low-dose dose ct scan' OR 'low-dose radiation computed tomography'/exp OR 'low-dose radiation computed tomography' OR 'low-dose radiation ct'/exp OR 'low-dose radiation ct' OR 'low-dose radiation ct scan'/exp OR 'low-dose radiation ct scan' OR 'low-radiation dose computed tomography'/exp OR 'low-radiation dose computed tomography' OR 'low-radiation dose computed tomography (low-dose ct)'/exp OR 'low-radiation dose computed tomography (low-dose ct)' OR 'low-radiation dose ct'/exp OR 'low-radiation dose ct' OR 'low-radiation dose ct scan'/exp OR 'low-radiation dose ct scan' OR 'low-dose computed tomography'/exp OR 'low-dose computed tomography') AND ('asia'/exp OR 'asia' OR 'asian*' OR 'china'/exp OR 'china' OR 'chinese'/exp OR 'chinese' OR 'korea'/exp OR 'korea' OR 'korean*' OR 'japan'/exp OR 'japan' OR 'japanese'/exp OR 'japanese' OR 'singapore*' OR 'india'/exp OR 'india' OR 'indian*') AND ('participant' OR 'participant s' OR 'participants' OR 'participate' OR 'participated' OR 'participates' OR 'participating' OR 'participation'/exp OR 'participation' OR 'participations' OR 'participative' OR 'participator' OR 'participators') AND [2011-2025]/py |
| Web of science | (TS=(lung neoplasms OR "neoplasms pulmonary" OR "neoplasm pulmonary" OR "Pulmonary Neoplasm" OR "Pulmonary Neoplasms" OR "neoplasms lung" OR "Lung Neoplasm" OR "neoplasm lung" OR "Lung Cancer" OR "cancer lung" OR "cancers lung" OR "Lung Cancers" OR "Cancer of Lung" OR "Pulmonary Cancer" OR "cancer pulmonary" OR "cancers pulmonary" OR "Pulmonary Cancers" OR "Cancer of the Lung")) AND (TS=("early detection of cancer" OR "Cancer Early Detection" OR "Early Diagnosis of Cancer" OR "Cancer Early Diagnosis" OR "Cancer Screening" OR "screening cancer" OR "Cancer Screening Tests" OR "Cancer Screening Test" OR "screening tests cancer" OR "test cancer screening" OR "tests cancer screening")) AND (TS=("low-dose computed tomography" OR "low-dose computer tomography" OR "low-dose CT" OR "low-dose radiation CT" OR "low-radiation dose CT" OR "low-dose computed tomography" OR "LDCT")) AND (TS=(Asia OR Asian* OR China OR Chinese OR Korea OR Korean* OR Japan OR Japanese OR Singapore* OR India OR Indian*) AND TS=(participants OR participate OR participation))with publication date from Jan 2011 to Oct 2025 |

| **Table S2.** The Newcastle-Ottawa Scale for risk assessment. | | |
| --- | --- | --- |
| Category | Item | Scale |
| Selection | 1) Representativeness of the Exposed Cohort | a) Truly representative of the average population in the community *  b) Somewhat representative of the average population in the community *  c) Selected group of users  d) No description of the derivation of cohort |
|  | 2) Selection of the non-exposed cohort | Irrelevant |
|  | 3) Ascertainment of exposure | a) Secure record (e.g., medical records) *  b) Structured interview *  c) Written self-report  d) No description |
|  | 4) Demonstration that outcome of interest was not present at start of study | a) Yes *  b) No |
| Comparability | 1) Comparability of cohorts on the basis of the design or analysis | Irrelevant |
| Outcome | 1) Assessment of outcome | a) Independent blind assessment *  b) Record linkage *  c) Self-report  d) No description |
|  | 2) Was follow-up long enough for outcomes to occur | a) Yes *  b) No |
|  | 3) Adequacy of follow up of cohorts | Irrelevant |
| *A star is awarded. | | |

| **Table S3.** Quality assessment result. | | | | | | | | |
| --- | --- | --- | --- | --- | --- | --- | --- | --- |
| Study | Item 1 | Item 2 | Item 3 | Item 4 | Item 5 | Item 6 | Item 7 | Item 8 |
| A.Panina et al,2022 | * | / | * |  | / | * | * | / |
| C.Wang et al,2023 | * | / | * |  | / | * | * | / |
| D.H.Wei et al,2021 | * | / | * | * | / | * | * | / |
| D.Liang et al,2020 | * | / |  |  | / | * |  | / |
| D.Liang et al,2021 | * | / | * |  | / | * | * | / |
| F.Zeng et al,2024 | * | / | * | * | / | * | * | / |
| H.Du et al,2022 | * | / | * |  | / | * | * | / |
| H.F.Xiao et al,2022 | * | / | * | * | / | * | * | / |
| H.Xiao et al,2023 | * | / | * |  | / | * | * | / |
| J.Du et al,2018 | * | / |  | * | / | * | * | / |
| J.Lee et al,2018 | * | / | * | * | / | * |  | / |
| J.Pan et al.2024 | * | / |  | * | / | * |  | / |
| J.Ren et al,2021 | * | / | * | * | / | * | * | / |
| J.Y.Zhu et al,2016 | * | / |  | * | / | * | * | / |
| L.W.Guo et al,2023 | * | / | * | * | / | * | * | / |
| L.Wang et al,2023 | * | / | * | * | / | * | * | / |
| L.Yang et al,2021 | * | / |  | * | / | * | * | / |
| M.J.Liu et al,2025 | * | / |  | * | / | * | * | / |
| N.Li et al,2022 | * | / | * | * | / | * | * | / |
| Q.Yang et al,2022 | * | / | * | * | / | * | * | / |
| S.L. Zhao et al,2025 | * | / |  | * | / | * | * | / |
| T.Tian et al,2022 | * | / |  | * | / | * | * | / |
| Thuy Linh Duong et al,2021 | * | / |  | * | / | * |  | / |
| W.Cao et al,2022 | * | / | * | * | / | * | * | / |
| W.J.Tao et al,2024 | * | / | * | * | / | * |  | / |
| W.Q.Chen et al,2020 | * | / |  | * | / | * | * | / |
| X.Y.Gu et al,2017 | * | / |  | * | / | * | * | / |
| X.Zhang et al,2024 | * | / | * | * | / | * | * | / |
| Y.J.Li et al.2021 | * | / | * | * | / | * |  | / |
| Y.P.Lin et al,2019 | * | / | * | * | / | * | * | / |
| Y.S.Zhang et al,2022 | * | / |  | * | / | * | * | / |
| Y.Wen et al,2021 | * | / | * | * | / | * | * | / |
| Y.Z.Liu et al,2018 | * | / |  | * | / | * | * | / |
| Z.F.Yu et al,2020 | * | / | * | * | / | * | * | / |
| Z.Yu et al,2023 | * | / | * | * | / | * | * | / |

**Table S4.** MOOSE checklist.

| **Item No** | **Recommendation** | **Reported**  **on Page No** |
| --- | --- | --- |
| Reporting of background should include | | |
| 1 | Problem definition | Page 4 |
| 2 | Hypothesis statement | Page 4 |
| 3 | Description of study outcome(s) | Page 5 |
| 4 | Type of exposure or intervention used | Page 5 |
| 5 | Type of study designs used | Page 5 |
| 6 | Study population | Page 5 |
| Reporting of search strategy should include | | |
| 7 | Qualifications of searchers (eg, librarians and investigators) | Page 6 |
| 8 | Search strategy, including time period included in the synthesis and key words | Page 5 |
| 9 | Effort to include all available studies, including contact with authors | Page 6 |
| 10 | Databases and registries searched | Page 5 |
| 11 | Search software used, name and version, including special features used (eg, explosion) | Page 5 |
| 12 | Use of hand searching (eg, reference lists of obtained articles) | Page 5 |
| 13 | List of citations located and those excluded, including justification | Page 5-6 |
| 14 | Method of addressing articles published in languages other than English | Page 5 |
| 15 | Method of handling abstracts and unpublished studies | Page 5-6 |
| 16 | Description of any contact with authors | No |
| Reporting of methods should include | | |
| 17 | Description of relevance or appropriateness of studies assembled for assessing the hypothesis to be tested | Page 8-9 |
| 18 | Rationale for the selection and coding of data (eg, sound clinical principles or convenience) | Page 6-7 |
| 19 | Documentation of how data were classified and coded (eg, multiple raters, blinding and interrater reliability) | Page 6-7 |
| 20 | Assessment of confounding (eg, comparability of cases and controls in studies where appropriate) | Page 7 |
| 21 | Assessment of study quality, including blinding of quality assessors, stratification or regression on possible predictors of study results | Page 7 |
| 22 | Assessment of heterogeneity | Page 7 |
| 23 | Description of statistical methods (eg, complete description of fixed or random effects models, justification of whether the chosen models account for predictors of study results, dose-response models, or cumulative meta-analysis) in sufficient detail to be replicated | Page 7-8 |
| 24 | Provision of appropriate tables and graphics | Figure 2-3, Table 2, Figure S1-6 |
| Reporting of results should include | | |
| 25 | Graphic summarizing individual study estimates and overall estimate | Figure 1-3 |
| 26 | Table giving descriptive information for each study included | Table 1 |
| 27 | Results of sensitivity testing (eg, subgroup analysis) | Page 9-10 |
| 28 | Indication of statistical uncertainty of findings | Page 9 |
| Reporting of discussion should include | | |
| 29 | Quantitative assessment of bias (eg, publication bias) | Page 8-9, Figure S1 |
| 30 | Justification for exclusion (eg, exclusion of non-English language citations) | Page 5-6 |
| 31 | Assessment of quality of included studies | Page 8-9, Table S3 |
| Reporting of conclusions should include | | |
| 32 | Consideration of alternative explanations for observed results | Page 15 |
| 33 | Generalization of the conclusions (ie, appropriate for the data presented and within the domain of the literature review) | Page 15 |
| 34 | Guidelines for future research | Page 15 |
| 35 | Disclosure of funding source | Page 16 |

| **Table S5.** List of included studies |
| --- |
| 1. Panina A, Kaidarova D, Zholdybay Z, Ainakulova A, Amankulov J, Toleshbayev D, et al. Lung Cancer Screening With Low-dose Chest Computed Tomography: Experience From Radon-contaminated Regions in Kazakhstan. J Prev Med Public Health. 2022 May;55(3):273–9.  2. Wang C, Liu M, He J, Hu M, Zhu J, Huang F, et al. Analysis of influencing factors on compliance of free low-dose omputed tomography screening among high-risk population of lung cancer in the community of Ma’anshan City. Anhui J Prev Med. 2023;29(2):94–9, 129.  3. Wei D, Ma Y, Zhang J, Yin H, Zhang X, Zhang Y, et al. Analysis of lung cancer screening rate and screening results of urban residents in Hefei from 2014 to 2016. Pract Oncol J. 2021;35(3):231–6.  4. Liang D, Hu J, Gao W, Shi J, Ma X, Xia C, et al. Analysis of lung cancer screening of urban residents  in Hebei province, 2018-2019. CHIN J CANCER PREV TREAT. 2020;27(22):1777–81.  5. Liang D, Shi J, Li D, Wu S, Jin J, He Y. Participation and Yield of a Lung Cancer Screening Program in Hebei, China. Front Oncol. 2022 Jan 10;11:795528.  6. Zeng F, Wang X, Wang C, Zhang Y, Fu D, Wang X. Analysis of screening outcomes and factors influencing compliance among community-based lung cancer high-risk population in Nanchang, China, 2018-2020. Front Oncol. 2024 Feb 9;14:1339036.  7. Du H, Wang X, Yu L. Analysis of risk assessment and screening results of lung cancer among residents in 3 cities of Shanxi Province from 2014 to 2018. Cancer Research and Clinic. 2022 Dec 28;34(12):925–9.  8. Xiao H, Yan S, Li J, Shi Z, Zou Y, Xu K, et al. Screening results and compliance analysis of low - dose spiral CT for lung cancer in Hunan from 2017 to 2018. Pract Oncol J. 2022;36(2):99–104.  9. Xiao H, Shi Z, Zou Y, Xu K, Yu X, Wen L, et al. One-off low-dose CT screening of positive nodules in lung cancer: A prospective community-based cohort study. Lung Cancer. 2023 Mar;177:1–10.  10. Du J, He M, Qiu, Lei H, Zhang Y, Zhang W, et al. Results of Lung Cancer Screening Among Urban Residents in Chongqing,2012~2017. China Cancer. 2018;27(5):328–32.  11. Lee JW, Kim HY, Goo JM, Kim EY, Lee SJ, Kim TJ, et al. Radiological Report of Pilot Study for the Korean Lung Cancer Screening (K-LUCAS) Project: Feasibility of Implementing Lung Imaging Reporting and Data System. Korean J Radiol. 2018;19(4):803–8.  12. Pan J, Wang J, Tao W, Wang C, Lin X, Wang X, et al. Is low-dose computed tomography for lung cancer screening conveniently accessible in China? A spatial analysis based on cross-sectional survey. BMC Cancer. 2024 Mar 14;24:342.  13. Ren J, Wang XB, Shu H, Xiong WJ, Wei QF, Wang X, et al. [Analysis of screening results and risk factors of high-risk populations of lung cancer in Nanchang city from 2018 to 2019]. Zhonghua Zhong Liu Za Zhi. 2021 Dec 23;43(12):1316–21.  14. Zhu J, Fan Y, Gu X, Gu X, Wen Z, Zhu L. An Analysis of Lung Cancer Screening with Low-Dose Computed Tomography for High -Risk Population in Urumqi Community. China Cancer. 2016;25(6):430–2.  15. Guo L, Meng Q, Zheng L, Chen Q, Liu Y, Xu H, et al. Special issue “The advance of solid tumor research in China”: Participants with a family history of cancer have a higher participation rate in low‐dose computed tomography for lung cancer screening. Int J Cancer. 2023 Jan 1;152(1):7–14.  16. Wang L, Wang Y, Wang F, Gao Y, Fang Z, Gong W, et al. Disparity in Lung Cancer Screening Among Smokers and Nonsmokers in China: Prospective Cohort Study. JMIR Public Health Surveill. 2023 Mar 14;9:e43586.  17. Yang L, Zhang X, Liu S, Li H, Li Q, Wang N, et al. Lung cancer screening in urban Beijing from 2014 to 2019. Chin J Prev Med. 2021 Mar 6;55(3):339–45.  18. Liu M, Huang S, Yu Z, Dai L, Xiang J, Qu Y, et al. Assessing factors influencing participation in LDCT lung cancer screening among high-risk urban populations in Nanjing, China. BMC Cancer. 2025 July 21;25:1196.  19. Li N, Tan F, Chen W, Dai M, Wang F, Shen S, et al. One-off low-dose CT for lung cancer screening in China: a multicentre, population-based, prospective cohort study. Lancet Respir Med. 2022 Apr;10(4):378–91.  20. Yang Q, Liu D, Lou P, Kong Y, Dong Z, Zhang P, et al. Results and cost-effectiveness analysis of lung cancer screening for urban residents in Xuzhou from 2014 to 2019. CHIN J CANCER PREV TREAT. 2022;29(7):463–7.  21. Zhao S, Li B, Yu Z, Du J, Zhou H, He M. Individual and Structural Factors Influencing Participation to Low-Dose Computed Tomography Screening in a Chinese Centralized Lung Cancer Screening Cohort. Arch Bronconeumol. 2025 Feb 1;61(2):96–100.  22. Tian T, Wei D, Xu Y, Xu K, Zhang Z, Qian L. Analysis of lung cancer screening f or urban residents in Hefei from 2015 to 2019. CHIN J CANCER PREV TREAT. 2022;29(23):1653–8.  23. Duong TL, Lee N, Kim Y, Kim Y. Assessment of the fear of COVID-19 and its impact on lung cancer screening participation among the Korean general population. Transl Lung Cancer Res. 2021 Dec;10(12):4403–13.  24. Cao W, Tan F, Liu K, Wu Z, Wang F, Yu Y, et al. Uptake of lung cancer screening with low-dose computed tomography in China: A multi-centre population-based study. eClinicalMedicine. 2022 July 29;52:101594.  25. Tao W, Yu X, Shao J, Li R, Weimin Li. Telemedicine-Enhanced Lung Cancer Screening Using Mobile Computed Tomography Unit with Remote Artificial Intelligence Assistance in Underserved Communities: Initial Results of a Population Cohort Study in Western China. Telemedicine and e-Health. 2024 June;30(6):e1695–704.  26. Chen W, Li N, Cao M, Ren J, Shi J, Chen H, et al. Preliminary Analysis of Cancer Screening Program in Urban China from 2013 to 2017. China Cancer. 2020;29(1):1–6.  27. Gu X, Gu X, Zhu J, Zhou T, Zhu L. Analysis of lung cancer screening results of 9265 urban residents in Urumqi from year 2014 to 2016. Pract Oncol J. 2017;31(3):242–5.  28. Zhang X, Hua J, Fan K, Wang D, Su L, Liu W, et al. Results of Lung Cancer Screening Among Urban Residents Aged 45~74 Years Old in Haikou City from 2019 to 2021 and Cost-Effectiveness Analysis. China Cancer. 2024;33(6):492–7.  29. Li Y, Du Y, Huang Y, Zhao Y, Sidorenkov G, Vonder M, et al. Community-based lung cancer screening by low-dose computed tomography in China: First round results and a meta-analysis. Eur J Radiol. 2021 Nov;144:109988.  30. Lin Y, Ma J, Feng J, Zhang Q, Huang Y. Results of Lung Cancer Screening among Urban Residents in Kunming. Chin J Lung Cancer. 2019;22(7):413–8.  31. Zhang Y, Lu G, Zhong H, Gao J. Screening compliance and screening results in high‐risk populations of lung cancer in Guangzhou. South China J Prev Med. 2022;48(12):1455–9.  32. Wen Y, Yu L, Du L, Wei D, Liu Y, Yang Z, et al. Analysis of low‐dose computed tomography compliance and related factors among high‐risk population of lung cancer in three provinces participating in the cancer screening program in urban China. Chin J Prev Med. 2021;55:633–9.  33. Liu Y, Kong Y, Luo X, Dong D, Chen H, Li N, et al. Analysis of early diagnosis and treatment of cancer in urban populations of Xuzhou city from 2014 to 2016. Jiangsu J Prev Med. 2018;29(4):382–5, 388.  34. Yu Z, Wang X, He R, Ren Y, Zhang L, Yan J, et al. Results of lung cancer screening in urban residents  in Liaoning Area, 2012 - 2017. Mod Prev Med. 2020;47(13):2442–5, 2470.  35. Yu Z, Ni P, Yu H, Zuo T, Liu Y, Wang D. Effectiveness of a single low-dose computed tomography screening for lung cancer: A population-based perspective cohort study in China. International Journal of Cancer. 2024 Feb 15;154(4):659–69. |
